# Modeling of non-additive mixture properties using the Online CHEmical database and Modeling environment (OCHEM)

**DOI:** 10.1186/1758-2946-5-4

**Published:** 2013-01-15

**Authors:** Ioana Oprisiu, Sergii Novotarskyi, Igor V Tetko

**Affiliations:** 1 Institute of Structural Biology, Helmholtz-Zentrum München – German Research Center for Environmental Health, Ingolstaedter Landstrasse 1b. 60w, D-85764 Neuherberg, Germany; 2 eADMET GmbH, Ingolstädter Landstraße 1, 60w, 85764 Neuherberg, Germany

## Abstract

The Online Chemical Modeling Environment (OCHEM, http://ochem.eu) is a web-based platform that provides tools for automation of typical steps necessary to create a predictive QSAR/QSPR model. The platform consists of two major subsystems: a database of experimental measurements and a modeling framework. So far, OCHEM has been limited to the processing of individual compounds. In this work, we extended OCHEM with a new ability to store and model properties of binary non-additive mixtures. The developed system is publicly accessible, meaning that any user on the Web can store new data for binary mixtures and develop models to predict their non-additive properties.

The database already contains almost 10,000 data points for the density, bubble point, and azeotropic behavior of binary mixtures. For these data, we developed models for both qualitative (azeotrope/zeotrope) and quantitative endpoints (density and bubble points) using different learning methods and specially developed descriptors for mixtures. The prediction performance of the models was similar to or more accurate than results reported in previous studies. Thus, we have developed and made publicly available a powerful system for modeling mixtures of chemical compounds on the Web.

## Background

Generally, QSPR (Quantitative Structure Property Relationship) models are limited to predicting properties of pure compounds. However, in recent years several studies have attempted to develop QSPR models to predict non-additive properties (density [1], infinite dilution activity coefficient [2], bubble temperature [3], azeotropic behavior [4], or excess molar volume [5]) of mixtures. There is also very considerable interest in modeling the toxicity of chemical mixtures.

The most challenging problem in QSARs of mixtures is representing a mixture by chemical descriptors. Therefore, prior to modeling, investigators should decide which descriptors are appropriate for such modeling. A further question concerns the proper external validation of models for mixtures, which is less obvious than in classical QSAR.

In our previous work [3,6] we developed new types of descriptors, based on classical (single compound) descriptors derived from the mixture components. Further, we analyzed two different strategies of model validation: a “mixtures out” and a “compounds out” approach. However, the development of models for mixtures requires very substantial effort regarding the storage and representation of mixtures, calculation of descriptors, and implementation of correct validation protocols. Thus, in our previous work modeling of mixtures was limited to only two sets of fragment-like descriptors, namely ISIDA fragments [7] and simplex descriptors [8], plus a few machine-learning approaches.

In order to overcome these limitations, new features were implemented in OCHEM [9] that allow reading and uploading of data for mixtures, creating special descriptors for mixtures, and validating models. Further, data for binary mixtures were collected from different sources [1,3,4] and analyzed to demonstrate the usefulness of the new tool. The developed models for several target properties are publicly available at http://ochem.eu/article/23416. The main goal of the project was to develop a web-based public resource for storage and analysis of chemical mixtures. Further, we have demonstrated that the previously proposed methodology for calculating descriptors of mixtures using fragment-based descriptors is easily extendable and can provide high accuracy models for other types of descriptors.

### Descriptors for mixtures

**Figure 1:**
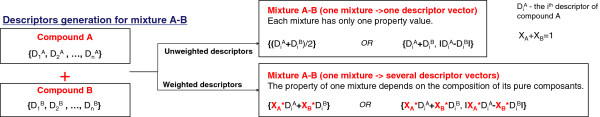
Methodology used to calculate descriptors for mixtures.

Mixture descriptors developed in this study were constructed as suggested in previous work [3,6], based on the descriptors of individual components of the mixture. Figure 1 shows two different descriptor types developed according to the modeled property.

(i) When the value of a mixture’s property does not depend on the concentration of its components, mixture descriptors are obtained by a simple (unweighted) average or sum and the absolute difference of the descriptor values corresponding to the individual constituents of the mixture. These mixture descriptors were successfully used to predict the azeotropic behavior of binary mixtures in a previous study [4] as well as in our work. It should be noted that each mixture corresponds to one property value.

(ii) When the value of the mixture property changes with the concentration of its components, mixture descriptors are calculated as mole-weighted sums or weighted sums and weighted absolute differences, using the descriptor value and mole fraction of each pure component in the mixture. These mixture descriptors have been used to predict the density and bubble point of binary mixtures in previous studies [1,3] as well as in our work. It should be noted that in this case each mixture is defined by several points (several property values) corresponding to the different concentration values of its components.

In our previous work, both types of mixture descriptors were based exclusively on the ISIDA fragment descriptors. In the current study, we extended this approach to arbitrary descriptor types.

### Validation protocol

As in classical QSAR, a rigorous external validation protocol is required to estimate the modeling of mixtures. However, the conventional external cross-validation procedure, where the points (compounds) are randomly placed in the external set (or fold), is unacceptable. This results in overestimation of the predictive performances of the developed models, especially when mixtures of the same compounds with different ratios are present several times in the dataset. Indeed, if both training and external sets include data points corresponding to the same mixture, the true predictive performance of a model will not be estimated properly.

**Figure 2:**
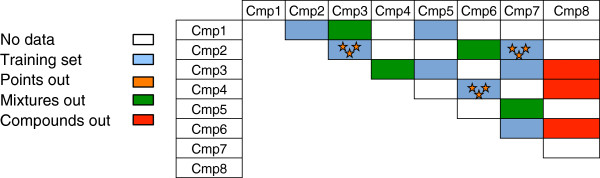
Protocols for validation of mixture property models.

Rigorous protocols for external validation were developed for QSAR modeling of mixtures and are presented in [3,6]. They involve three different strategies (Figure 2):

• *“Points out”*: data points are randomly placed in each fold of the external cross-validation set. Each mixture is present simultaneously in both the training and the external sets. This is the weakest validation protocol.

• “*Mixtures out*”: All data points corresponding to mixtures composed of the same constituents, but in different ratios, are simultaneously removed and placed in the same external fold. Thus, every mixture is present in either the training or the external set, but never in both sets.

• “*Compounds out*”: Pure compounds and their mixtures are simultaneously placed in the same external fold. Thus, every mixture in the external set contains at least one compound that is absent from the training set. This is the most rigorous validation protocol.

The “*points out*” strategy reflects the ability of models to predict existing mixtures with novel compositions and is rather limited. The second protocol evaluates the prediction performance of a model for new mixtures, while the “*compounds out*” protocol does so for new compounds. Therefore, only the “*mixtures out*” and “*compounds out*” strategies were used in the current study.

## Implementation

### Integration with the Online Chemical Modeling Environment

OCHEM is a user-friendly tool allowing any user on the Web to upload new data and develop QSAR models as well as access data and models published by others. The experimental data stored in the database can be easily manipulated to build predictable QSAR models using different machine-learning techniques (e.g., neural networks, support vector machines, random forest, etc.).

Prior to the current work, OCHEM was limited to the analysis of individual compounds. Based on knowledge of mixture predictions, new features were incorporated into OCHEM allowing users to store and model properties of binary non-additive mixtures.

#### Data format

A support for storing mixture data in the OCHEM database was developed. To do so, an Excel file is required containing the necessary information for mixtures. Each data point is represented by a row in the file. This must contain the structure (e.g., SMILES or SDF) of the compound with the largest molar fraction in the mixture, its molar fraction, and the molecular ID or structure (SMILES or SDF) corresponding to the second pure compound in the OCHEM database. The experimental value of the mixture property, its unit, and publication source should be also provided. An example of the Excel file required to upload mixtures is available on the Wiki website at http://wiki.ochem.eu/w/Upload_of_mixture_data.

The first compound in the binary mixture is always the one with the highest molar fraction. Thus, the molar fraction values reported in the database range between 0.5 and 1. It should be noted that for a mixture the sum of the molar fractions of its components always equal 1. Therefore, the value of the molar fraction of the second compound in a binary mixture can be easily obtained when the molar fraction of the first compound is known.

Users can also specify the name of the second compound in the mixture. In cases where the molar faction is <0.5, the first and second compound are interchanged and the complement to 1 used as the molar fraction. This procedure allows one to avoid duplicates when uploading mixtures.

The OCHEM record refers to one compound only. In order to allow calculation of descriptors for mixtures, the second compound should be also present. This poses a challenging problem for the design of the descriptor calculation procedure. We solved this by requiring all compounds with a molar fraction >0.5 to be present in the training set at least once, i.e., as a first compound in the mixture record, and/or they should be included with their pure properties. Indeed, the properties of pure compounds are always more easily accessible. For all the studies reported below, we extended the datasets with pure compounds.

#### Descriptors and validation protocol

As mentioned in section 2.1, special descriptors for mixtures were constructed based on those computed for the individual compounds constituting the mixture. Users can choose between four different descriptor types, obtained by simple averaging of the descriptors, the sum and absolute difference of the descriptors, the weighted sum of the descriptors, or the weighted sum and weighted absolute difference of the descriptors.

**Figure 3:**
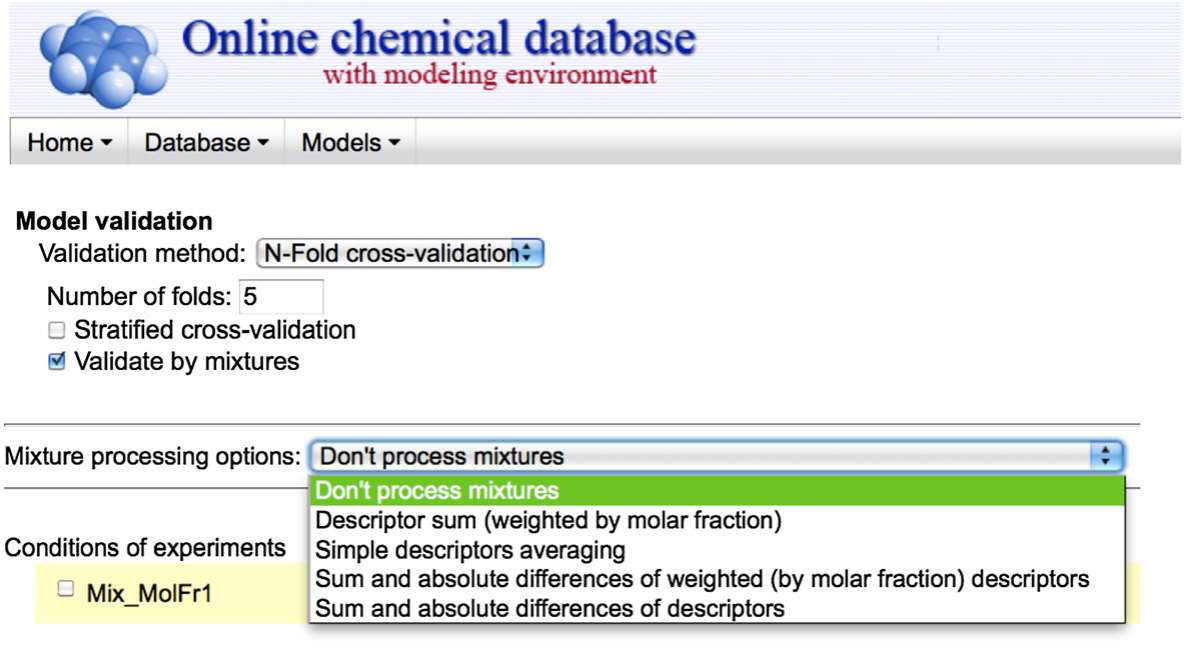
Screenshot of the OCHEM features implemented to analyze mixtures.

Further, two different validation types were implemented: “*mixtures out*” and “*compounds out*.” The default validation type was “*compounds out*”, i.e., the most rigorous validation protocol. Figure 3 shows a screenshot of the OCHEM Web interface giving the mixture calculation options.

## Results and discussion

### Modeling properties of binary mixtures

Qualitative and quantitative models were developed using different learning methods and different sets of descriptors, implemented in OCHEM. In this section, we report the performances of the obtained models and then compare them with models developed in previous studies.

### Density

Ajmani et al. studied the density of binary mixtures in [1]. In their study, the QSPR methodology was applied to 4679 data points of experimentally measured densities of binary liquid mixtures compiled from the literature, corresponding to 271 binary mixtures. QSPR models were developed to predict the deviation of the experimental mixture density from the “ideal” mixture density, calculated by combining the densities of the single components according to their ratio in the mixture. Two methods of training/test set creation were used, QMD-1 and QMD-2, which correspond respectively to the “*points out*” and “*mixtures out*” validation strategies (see section 2.2). In addition, the authors used 39 of the 271 mixtures (for the QMD-2 strategy) for external validation.

We found that the authors did not notice eight mixture duplicates (in total 144 data points), which are shown in Table S1 (see Additional file 1: Table S1). Some of these duplicates were between training and test sets. Such duplicates could bias the statistical results of the models for both strategies reported in the study. After eliminating duplicates, the training set contained 3734 data points (we also included 124 pure compounds) and 672 for the test set.

Our first analysis was to attempt to reproduce the authors’ model developed using seven Dragon descriptors, namely EEig04d, BEHv3, Mor15m, H0e, GATS1e, E2e, and Mor10e. In their original work the authors calculate these descriptors using E-Dragon, which is supported by the Virtual Computational Chemistry Laboratory (VCCLAB [10]). The VCCLAB used Dragon version 5.4 [11], which is also available on OCHEM. The authors used a simple averaging of descriptors according to their molar fraction. The same descriptors were used to develop a neural network model using the ASNN method with all default parameters as provided by the OCHEM website. The developed model predicted test set molecules with a squared Pearson correlation coefficient (R^2^) of 0.73 ± 0.04 and a root mean squared error (RMSE) of 0.014 ± 0.001. These coefficients are lower than those (R^2^ = 0.86, RMSE = 0.0091) reported in Ajmani et al. for the same descriptors. The difference could originate from for instance the preparation of chemical structures, the machine-learning approaches used, or the presence of duplicated structures. For example, we did not optimize structures and simply used 2D to 3D conversion using Corina. However, with the full set of Dragon descriptors, ASNN calculated an accuracy of R^2^ = 0.85 ± 0.04, RMSE = 0.0099 ± 0.0006, which was similar to the aforementioned result reported by Ajmani et al.

Similar performances for the prediction of the test set compounds were calculated for different methods and algorithms using a Comprehensive Modeling (CM) feature of OCHEM. With a few clicks, CM enables users to explore data using different descriptors and machine-learning approaches. The models with the highest accuracy according to “*compounds out*” 10-fold cross-validation are summarized in Table 1. Both “*mixtures out*” and “*compounds out*” 10-fold cross-validation results are given.

**Table 1:** **Statistical parameters of Online Chemical Modeling Environment (OCHEM,**http://ochem.eu**) models with the lowest RMSE according to the “*compounds-out*” cross-validation protocol for the prediction of binary mixture densities**

	**“*Compounds out*” validation**		**“*Mixtures out*” validation**		**Test set prediction (“*compounds out*” validation)**	
Method/descriptors	R^2^	RMSE	R^2^	RMSE	R^2^	RMSE
LibSVM/Dragon	0.69 ± 0.05*	0.014 ± 0.001	0.81 ± 0.09	0.011 ± 0.002	0.88 ± 0.04	0.0089 ± 0.001
ASNN/Inductive descriptors [12]	0.68 ± 0.04	0.014 ± 0.0008	0.72 ± 0.04	0.0131 ± 0.0009	0.81 ± 0.06	0.011 ± 0.001
LibSVM/Inductive descriptors	0.71 ± 0.05	0.014 ± 0.001	0.81 ± 0.03	0.0109 ± 0.0005	0.88 ± 0.04	0.0084 ± 0.001
ASNN/Dragon [11]	0.56 ± 0.06	0.016 ± 0.001	0.69 ± 0.04	0.014 ± 0.001	0.85 ± 0.04	0.0099 ± 0.001
ASNN/ChemAxon [13]	0.55 ± 0.06	0.017 ± 0.001	0.69 ± 0.04	0.0137 ± 0.0009	0.88 ± 0.03	0.0088 ± 0.001

* 95% confidence intervals were calculated using a bootstrap procedure based on 1,000 replicas (implemented for all models in OCHEM). RMSE – Root Mean Squared Error; R^2^ – square of the Pearson correlation coefficient.

As expected, the prediction performance estimated using the “*compounds out*” protocol is generally lower than that using “*mixtures out*” validation. Cross-validation results calculated using both protocols are lower (sometimes significantly so) compared to those calculated for the test set, which corresponds to the “*mixtures out*” validation. As stated by the authors, the test set of mixtures was generated using the Sphere exclusion method. Thus, it was selected to provide the best possible coverage of the space of analyzed mixtures. In practice, the developed models are expected to be applicable to arbitrary mixtures. Thus, it is likely that the performance of models for new data will be similar to that of n-fold cross-validation rather than to those obtained for the specifically designed validation set.

So far we have used descriptors calculated as mole-weighted sums of individual mixtures. OCHEM also calculates the second type of descriptors, that is, the weighted absolute difference of the descriptors. Using these descriptors, the results for the test set prediction calculated by LibSVM with ChemAxon descriptors were R^2^ = 0.94 ± 0.02, RMSE = 0.006 ± 0.001 – the highest prediction accuracy achieved for this test. However, the results of the same method, as well as of other methods for the “mixture-out” and “compound-out” validation protocols, are not significantly different from those calculated using the weighted sum of individual mixtures.

### Bubble temperature

Vapor–liquid equilibrium (VLE) data are one of the most important types of information for evaluation of the phase behavior of binary liquid mixtures, which is crucial for the design of separation processes [14]. Vapor–liquid equilibrium is a state where a liquid and its vapor are in equilibrium with one another, i.e., when the rate of evaporation equals the rate of condensation. A VLE curve shows the variation of the equilibrium composition of the liquid mixture with temperature, at fixed pressure. The dew-point curve represents the temperature at which the saturated vapor starts to condense, whereas the bubble point is the temperature at which the liquid starts to boil.

Bubble points of 167 mixtures containing 3232 data points were modeled by Oprisiu et al. [3]. An external test set of 94 mixtures containing almost 2000 data points was also used. Two versions of this set were used. For “compound-out” validation we used 67 mixtures, which included 34 new compounds (in total 1309 measurements); for “mixture-out” validation we used a set of 631 measurements, which included 27 new mixtures. In our previous work, we developed a consensus model using nonlinear Support Vector Machine (SVM), Associative Neural Networks (ASNN), and Random Forest (RF) approaches. For SVM and ASNN calculations, the ISIDA fragment descriptors were used, whereas Simplex descriptors were employed in RF models.

The same data were modeled with OCHEM using LibSVM machine-learning and the same Dragon descriptors. Table 2 shows that the performances of the developed models are similar to or higher than those from the previous work, which demonstrates the usefulness of the developed tool. Further, the results obtained in the previous study (ref [3]) were based on a consensus prediction of three models, namely ASNN, SVM, and RF. The LibSVM method achieved similar performance without needing to build a consensus model.

**Table 2:** **Comparison of performances of OCHEM models with the consensus model of Oprisiu et al.**[3]**for the prediction of bubble temperatures of mixtures**

		**“*Mixtures out*” validation**		***“Compounds out”***	
		**OCHEM**	**Ref [**[3]**]**	**OCHEM**	**Ref [**[3]**]**
Training set	Q^2^	0.93 ± 0.01	0.95	0.92 ± 0.03	0.9
	RMSE	6.2 ± 0.6	5.2	6.5 ± 0.5	7.0
Test set	Q^2^	0.92 ± 0.01	0.88	0.56 ± 0.06	0.4
	RMSE	5.7 ± 0.3	5.9	19 ± 1	21.4

Q^2^ – coefficient of determination.

### Azeotropic behavior

Azeotropic data are very important in the design of distillation processes, and their theoretical assessment could significantly reduce the costs of selection of proper agents for industrial processes. An azeotrope is a liquid mixture that boils at a constant temperature, keeping its composition fixed. When an azeotrope boils, the resulting vapor has the same component ratio as the liquid phase with which it is in equilibrium.

Classification models were developed using 400 mixtures (200 azeotropes/200 zeotropes) and validated on a data set of 95 mixtures containing only pure compounds already included in the training set.

The same data sets were used in OCHEM. The pure compounds (n = 65) of the mixtures were considered zeotropes and were included in the training set. Thus 465 mixtures were used as the training set and 95 for the test set.

Table 3 shows the classification results obtained with OCHEM, using the Weka Random Forest algorithm and Adriana descriptors. The performances of the obtained models are similar to those from previous work. OCHEM results calculated for mixture-out and compound-out protocols have similar accuracy.

**Table 3:** **Comparison of classification results for the azeotropic behavior of mixtures calculated using OCHEM and Oprisiu**[4]**models**

	5-Fold Cross Validation (400 mixtures)*			**Test set (95 mixtures)**	
	**OCHEM compound out**	**OCHEM mixture out**	**Oprisiu [**[4]**] mixture out**	**OCHEM mixture out**	**Oprisiu [**[4]**] mixture out**
Balanced Accuracy	0.80 ± 0.04	0.78 ± 0.04	0.82	0.85 ± 0.07	0.82
Recall of zeotrops	0.77	0.77	0.78	0.74	0.73
Recall of azeotropes	0.83	0.80	0.85	0.95	0.91

*OCHEM models were developed using 465 mixtures, which also included pure compounds.

## Conclusion

OCHEM was extended with tools to store and model binary mixtures. The theoretical part of the modeling approach was based on the PhD research of I.O. [3,4] as well as on previous studies [1].

We have developed an original way of storing and processing mixtures in the database. New descriptors for mixtures were used, based on classical (single compound) descriptors derived from the mixture components. In addition, specific cross-validation protocols for mixtures were implemented: “*mixtures out”* and “*compounds out*.” To validate our implementation, qualitative and quantitative models were developed with good predictive performance, using different learning methods and different sets of descriptors. The developed models are available at http://ochem.eu/article/23416 and can be used to predict properties of new mixtures.

The main purpose of this study was to contribute publicly available, web-based tools for the modeling and prediction of mixtures of chemical compounds. The availability of such tools will stimulate developments in this research area. Indeed, preparation of data, calculation of mixture descriptors, and application of the correct validation protocol require considerable effort and can be prone to error. The developed tools allow users to significantly simplify this procedure and all analyses can be performed with a few clicks. The mixtures database also inherits all the general features of OCHEM, namely strict control of the information source, storage of records in original units (and automatic conversion to units required for modeling), tracking of changes, public and hidden records, storage of experiment conditions, and automatic detection of duplicates. The modeling of mixtures provides automatic calculation of prediction accuracy, use of a wide spectrum of machine-learning algorithms and descriptors, and storage, publishing, and application of models.

In addition, while it was intuitive to propose descriptors for mixtures based on fragmental and simplex descriptors[1,3,4], the extension of this methodology to arbitrary descriptors was not obvious. We have shown that the same methodology can be applied successfully with different types of descriptors and different machine-learning methods, thus generalizing the previous findings.

The approach developed here can be used to model any other non-additive properties of binary mixtures, such as viscosity, toxicity, or antiviral activity. The methodology can be straightforwardly extended to multiple compound mixtures containing more than two compounds.

## Availability and requirements

The developed software is publicly available on-line from On-line CHEmical database and Modelling environment (OCHEM) platform http://ochem.eu. It can be accessed with any modern web browser, which supports Javascript. We recommend the latest version of Firefox http://www.mozilla.org/firefox.

## Competing interests

The publication costs were covered with MC ITN “Environmental ChemOinformatics” (ECO) project http://www.eco-itn.eu, which is scientifically coordinated by IVT. IO was ECO fellow. SN and IVT are shareholders and employees of eADMET GmbH which licences OCHEM http://ochem.eu platform.

## Authors’ contributions

IO programmed methods for mixture descriptor calculations, collected, uploaded and curated the mixture data, performed calculations and drafted the article. SN developed framework for storage and analysis of mixtures. IVT programmed cross-validation algorithms and supervised the project. All authors read and approved the final manuscript.

## Supplementary Material

Additional file 1: Table S1. Mixture duplicates found in the Ajmani [1] data set. The following additional data are included with the online version of this paper. Table S1 lists eight mixture duplicates (in total 144 data points) found in the Ajmani et al. density data for binary mixtures [1]. Click here for file
