# Supplementary material for: Modeling of non-additive mixture properties using the Online CHEmical database and Modeling environment (OCHEM)
Source: J Cheminform. 2013 Jan 15;5:4. doi: 10.1186/1758-2946-5-4 (PMC3568005; doi:10.1186/1758-2946-5-4)
Supplement: Additional file 1: Table S1. — Mixture duplicates found in the Ajmani [1] data set. The following additional data are included with the online version of this paper. Table S1 lists eight mixture duplicates (in total 144 data points) found in the Ajmani et al. density data for binary mixtures [1]. [file 1758-2946-5-4-S1.docx]

# Supplementary materials.

Table S1 Mixture duplicates found in the Ajmani[[1](#_ENREF_1)] data set.

| Num. | Mixture |
| --- | --- |
| 1. | Benzene+etylacetate |
| 2. | Methylmethacrylate+benzene |
| 3. | Cyclohexane+methylmetacrylate |
| 4. | Propiophenone+toluene |
| 5. | Methylmethacrylate+p-xylene |
| 6. | Water+acetone |
| 7. | Water+dimetylethanolamine |
| 8. | Water+diethylethanolamine |
